# Effects of mushroom waster medium and stalk residues on the growth performance and oxidative status in broilers

**DOI:** 10.5713/ajas.19.0889

**Published:** 2020-03-04

**Authors:** Y. C. Hsieh, W. C. Lin, W. Y. Chuang, M. H. Chen, S. C. Chang, T. T. Lee

**Affiliations:** 1 Department of Animal Science, National Chung Hsing University, Taichung, 402, Taiwan; 2 Taiwan Agricultural Research Institute, Council of Agriculture, Executive Yuan, Taichung City, 41362, Taiwan; 3 Kaohsiung Animal Propagation Station, Livestock Research Institute, Council of Agriculture, Executive Yuan, Pingtung, 91201, Taiwan; 4 The iEGG and Animal Biotechnology Center, National Chung Hsing University, Taichung, 402, Taiwan

**Keywords:** Mushroom, Waster Medium, Stalk Residues, Broiler Chickens, Antioxidant

## Abstract

**Objective:**

The study developed mushroom stalk residues as feed additives in the broiler diet for improving the growth performance and immunity of broilers as well as to increase the value of mushroom stalk residues.

**Methods:**

In total, 300 ROSS 308 broilers were randomly allocated into fifteen pens with five dietary treatments: i) control, basal diet; ii) CMWM, supplemented with 1% *Cordyceps militaris* waster medium (CM); iii) CMPE, supplemented with 0.5% CM+0.5% *Pleurotus eryngii* stalk residue (PE); iv) CMPS, supplemented with 0.5% CM+0.5% *Pleurotus sajor-caju* stalk residue (PS); v) CMFV, supplemented with 0.5% CM+0.5% *Fammulina velutipes* stalk residue (FV).

**Results:**

The chemical analysis results showed that CM extracts, PE extracts, PS extracts, and FV extracts contain functional components such as polysaccharides and phenols and have both 2, 2-diphenyl-1-picryl-hydrazyl-hydrate scavenging and Ferrous scavenging capacities. The group CMWM saw increased body weight gain and feed conversion rate and the promotion of jejunum villus growth, but there is no significant difference in the intestinal bacteria phase. Antioxidant genes in the nuclear factor (erythroid-derived 2)-like 2 (Nrf2)-antioxidant responsive element pathway among the groups are significantly higher than that of the control group, especially in group CMWM.

**Conclusion:**

The mushroom stalk residues have antioxidant functional components, can improve the intestinal health and body weight gain of chickens, and can activate the antioxidant pathway of Nrf2 to increase the heme oxygenase-1 expression. The treatment with 1% CM was the most promising as a feed additive.

## INTRODUCTION

Animal rearing environment will often affect animal health by crowding, temperature, and opportunistic infection with some microbes [1–4]. While these external pressures make animals use energy to resist the stress, the oxidative environment in their body also loses its balance and increases their oxidative stress, causing an oxidative chain reaction to destroy cell tissue [1]. In the face of these environmental challenges, people often choose to add trace antibiotics to animal feed, which can reduce the chance of inflammation in the intestine. If inflammation is prevented, then stress of the animal or the oxidative stress in its body will be indirectly reduced and can decrease energy lose, which can then be used in the immune system to keep it in a comfortable state and to increase productivity [5]. However, with the gradual ban of antibiotics in the EU in 2006, the world has begun to pay more and more attention to drug residues and drug-resistant bacteria. Therefore, the animal feed industry is looking for alternatives to antibiotics.

Some reactive oxygen species (ROS) can cause oxidative damage to animal organs through the properties of their free radicals [6]. Among the many functional components contained in mushrooms, like phenolic compounds, triterpenoids have strong anti-oxidation ability. Studies have shown that triterpenoids have the function of preventing oxidative damage caused by free radicals and can prevent or delay the onset of disease [7]. Some studies have also indicated that phenolic compounds have the ability to reduce lipid oxidation [8]. Previous studies found that *Cordyceps militaris* has components such as cordycepin, phenolic compounds, adenosine, and various polysaccharides [9], which are believed to activate macrophages via the nuclear factor-κB pathway and promote the performance of major histocompatibility complex molecules enhancing the ability of antigen presentation [10].

Studies have also shown that *Pleurotus eryngii* has many functional ingredients, such as phenols and polysaccharides, which have antioxidant properties and improve the meat quality of broilers [11]. *Pleurotus eryngii* can also improve the performance of laying hens as well as the egg quality and antioxidant capacity of eggs [12]. In addition, there are also references to bactericidal activity and serum immune responses [13]. *Pleurotus sajor-caju* has a similar nutritional composition to *Pleurotus ostreatus* and presents antioxidant activity [14]. *Pleurotus sajor-caju* stalk residue (PS) has also been shown to have antibacterial activity against gram-positive and gram-negative bacteria [15]. *Fammulina velutipes* is believed to be effective at improving the health of laying hens and has been shown to improve egg integrity and increase calcium in eggshells in laying hens [16].

After a culture is completed, the fruiting bodies are sold, but the culture medium and stalk residues are treated as waste. These white-rot fungus wastes have been proven to improve the production performance of laying hens [17]. Nevertheless, the literature has rarely compared the use of different stalk residues in feed. Thus, the purpose of this experiment is to investigate whether different combinations of mushroom stalk residues as feed additives can improve the health and performance of broilers.

## MATERIALS AND METHODS

The *Cordyceps militaris* waster medium (CM), *Pleurotus eryngii* stalk residues (PE), *Pleurotus sajor-caju* stalk residues (PS), and *Fammulina velutipes* stalk residues (FV) in this study were taken from Taiwan’s Agricultural Research Institute, COA. The mushroom stalk residues were extracted with distilled water (1:10, w/v) at 95°C for 2 hours and filtered with a filter paper (Advantec No. 1, Tokyo, Japan). The filtrate was used for subsequent analysis.

### 2, 2-Diphenyl-1-picryl-hydrazyl-hydrate radical scavenging capacity (%)

The experiment prepared Tris-Hcl (pH 4.7, 100 mM) buffer and ethanol solution containing 250 μM 2, 2-diphenyl-1-picryl-hydrazyl-hydrate (DPPH), mixed 100 μL samples of different concentrations and 400 μL of the above buffer, added 500 μL of the above DPPH solution, and let stand for 20 minutes in the dark. Absorbance was measured at 517 nm, and ethanol was used as a control. A lower absorbance value of the reaction mixture indicated a stronger scavenge effect and was calculated by DPPH scavenging effects (%) = (1−[A1/A0])×100%, in which A1 is the net absorbance in the presence of the sample (the sample measured absorbance - background absorbance), and A0 is the absorbance of the control reaction (ethanol) [18].

### Ferrous scavenging capacity (%)

The experiment prepared FeCl2 (ferrous chloride · 4H_2_O, 2 mM) and ferrozine (5 mM), added 0.25 mL of different concentrations of the sample to 0.8 mL of deionized water and 0.025 mL of 2 mM FeCl_2_, and then added 0.05 mL of 5 mM Ferrozine solution at room temperature for 30 seconds. After 10 minutes of reaction, the absorbance was measured at 562 nm, and 0.25 mL of deionized water was used as a control. A lower absorbance value of the reaction mixture indicated a stronger scavenge effect and was calculated by Ferrous ion chelating (%) = (1−[A1/A0])×100%, in which A1 is the net absorbance in the presence of the sample (the sample measured absorbance − background absorbance), and A0 is the absorbance of the control reaction (deionized water) [19].

### Animal experimental design

The experimental animals and breeding environment were approved by National Chung Hsing University, Taiwan (IACUC: 107-043). Three hundred ROSS 308 female broilers were selected and separated into five treatment groups: control group, 1% golden *Cordyceps militaris* waster medium group (CMWM), 0.5% CM + 0.5% PE group (CMPE), 0.5% CM + 0.5% PS group (CMPS), and 0.5% CM + 0.5% FV group (CMFV). Each treatment was repeated three times of 20 broilers per replications (a total of 60 birds/treatment). The broilers were kept in a temperature-controlled house, and the temperature was controlled at 27°C±2°C. The broilers within 1 to 7 day-old were kept warm by fences and heater lamps. The field was a concrete floor (2.5 m×4 m) with rice bran. Two feed drinkers and one feed tray were installed in each field in the first 21 days; the nipple drinker and feed trough were replaced every 22 to 35 days. A vaccine for Newcastle disease and infectious bronchitis was provided via the nose when the broilers were one day old. The form of feed formula was formulated according to the nutritional requirements of broilers [20] and contained no coccidia and antibiotics (Tables 1, 2). The lighting program was based on Ramlucken et al [21], the broilers were exposed to 23 h of light up to 7 D of age, and then from there onwards they received 8 h of darkness continuously in a 24-h period. The experimental materials and feed composition were analyzed according to AOAC [22].

### Performance and serum intestinal content collection

The experiment collected the weights of broilers at the ages of 1, 21, and 35 days, and feed consumption was recorded at 21 and 35 days of age. The calculated data included body weight gain, feed intake, and feed conversion rate (FCR) of the birds. At 35 days of age, 4 broilers were randomly selected from each cage, and 2 of them had 5 mL of blood collected from the wing vein by a heparin anticoagulation tube for detection of quantitative real-time polymerase chain reaction (RT-PCR). The other two broilers were euthanized by electric shock, and the contents of ileum and cecum were removed from the lower abdomen to detect intestinal flora.

### Microbial populations in ileal and cecal contents

The experiment collected fresh ileum and cecum contents, put 1 g of chyme into 9 mL deionized water, and measured the pH value by a pH meter. One g of chyme was put into 9 mL phosphate-buffered saline (PBS) and the sequence dilution for the microbial parameter. The amount of bacteria after dropping 0.1 mL of the bacteria into an appropriate medium and smearing them was counted. Coliform was cultured in aerobic 37°C for 24 hours with chromogenic medium agar (CHROagar ECC, Springfield, NJ, USA); *Lactobacillus* spp. were cultured at anaerobic 37°C for 48 hours with De Man, Rogosa and Sharpe agar (Difco Lactobacilli MRS Agar, Franklin Lakes NJ, USA); and *Clostridium perfringens* was cultured for 48 hours with tryptose sulfite cycloserine agar (GranuCult TSCagar, Merck, Kenilworth, NJ, USA) at anaerobic 37°C. After culturing, the number of colony-forming unit (CFU) on the agar was counted.

### Intestinal morphology

At the age of 35 days, 4 broilers were randomly selected from each column to be euthanized by electric shock. The jejunum (the bile duct junction to the Merkel ventricle) and the ileum (the Merkel ventricle to the cecum junction) were removed from the lower abdomen. The contents of the intestines were carefully washed away in 3 cm with a PBS solution, cut into small pieces, and placed in 10% formalin for fixation. After standing for 5 days, they were entrusted to the Department of Veterinary Medicine, National Chung Hsing University, Taiwan for paraffin embedding, sectioning, and staining. Intestinal sections were observed using an optical microscope, the villus height, crypt depth, and muscle layer thickness were measured with a Motic Image Plus 2.0 analysis system (Motic Instruments, Richmond, Canada), and the villus height and crypt depth ratio were calculated.

### Quantitative real-time polymerase chain reaction

After centrifuging fresh blood, it was aspirated with supernatant and PBS was added to the original volume. After slowly shaking, 3 mL of the mixture was pipetted into 3 mL of Ficol and centrifuged at 200 g for 30 minutes. A middle layer of peripheral blood mononuclear cell (PBMC) was taken to a new centrifuge tube and 3 mL of PBS was added; mix and centrifuge to remove the supernatant and scrape off the precipitated blood cells, add 3 mL of PBS, mix and centrifuge, and discard the supernatant. The number of precipitated blood cells was calculated and added back to the volume of PBS. Next, 20 μL of the dye was extracted, and the blood cells were mixed in equal amounts. After counting the number of cells, reconstituted the Roswell Park Memorial Institute (RPMI) medium and adjusted the number of cells to 1×10^7^ cells/mL. The mRNA in PBMC was extracted with a Direct-zol RNA miniprep kit and modified according to the method of Yamaguchi et al [23]. The mRNA was reverse transcribed into cDNA, and its concentration was measured by qPCR. B-actin was used as a standard for all genes and was calculated by the method of 2^−ΔΔCt^. All the primer sequences are shown in Table 3.

### Statistical analysis

All data were employed by SAS (SAS 9.4, 2018) statistical software and analyzed by analysis of variance for the completely randomized design using the general linear model program. Determination of differences between the mean values of the groups was conducted by Tukey. The differences between the treatments were compared by the p<0.05.

## RESULTS

### Component analysis

Table 4 presents the analysis of the chemical composition and the bioactive compounds of CM, PE, PS, and FV. CM contained 97.3% dry matter, 1.23% ash, 7.8% crude protein, 63.1% neutral detergent fiber (NDF), 8.0% acid detergent fiber (ADF) and 3.84% crude fat; PE contained 94.5% dry matter, 5.91% ash, 17.21% crude protein, 57.9% NDF, 7.4% ADF and 6.39% crude fat; PS contained 97.9% dry matter, 6.77% ash, 22.43% crude protein, 61.9% NDF, 8.2% ADF and 2.01% crude fat; FV contained 93.0% dry matter, 11.54% ash, 12.65% crude protein, 60.8% NDF, 8.0% ADF and 5.04% crude fat. The polysaccharide content of the CM was the highest, because the cultivation substrate was brown rice, which was much higher than other test materials. The content of total triterpenoids was highest in the content of FV, followed by PS and PE.

### 2, 2-Diphenyl-1-picryl-hydrazyl-hydrate radical scavenging capacity

**Figure 1:**
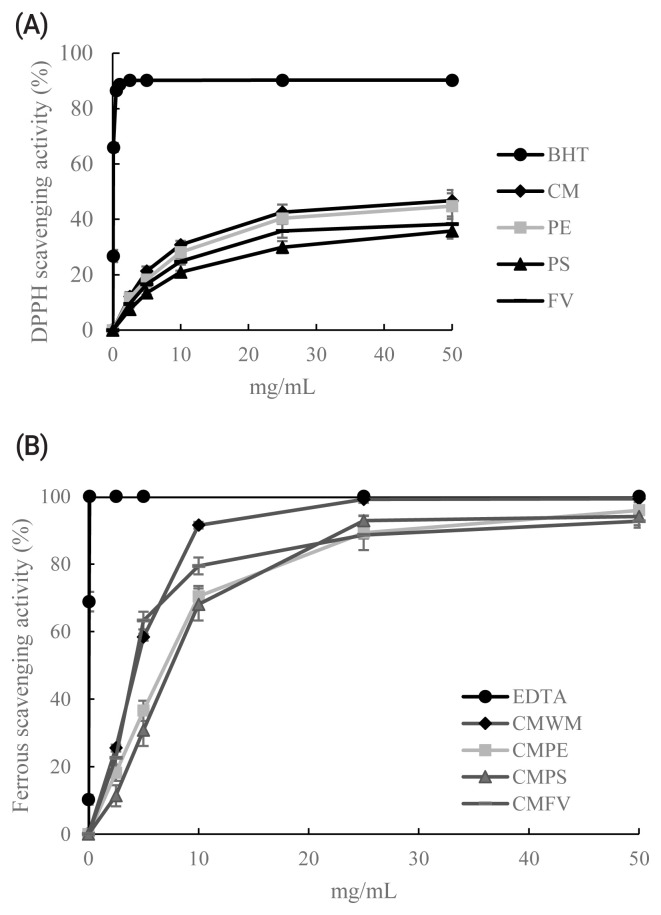
*In vitro* antioxidant assay of *Cordyceps militaris* waster medium (CM); *Pleurotus eryngii* stalk residues (PE); *Pleurotus sajor-caju* stalk residues (PS); *Fammulina velutipes* stalk residues (FV). (A) 2, 2-diphenyl-1-picryl-hydrazyl-hydrate (DPPH) scavenging capacity (B) Ferrous scavenging capacity. Values are mean±standard deviation.

The DPPH radicals’ scavenging capacity of four different mushroom waster medium or stalk residues are shown in Figure 1A. The scavenging capacity of the DPPH free radical was as good as that of butylated hydroxytoluene. The results show that DPPH radicals’ scavenging capacity of CM, PE, PS, and FV at 10 mg/mL reached 34.2%, 31.2%, 23.2%, and 27.5%, respectively.

### Ferrous scavenging capacity

The Ferrous scavenging capacity of four different mushroom waster medium or stalk residues are shown in Figure 1B. The Ferrous scavenging capacity was as good as that of ethylenediaminetetraacetic acid. The results show that the Ferrous scavenging capacity of CM, PE, PS, and FV at 10 mg/mL reached 91.5%, 70.4%, 68.0%, and 79.4%, respectively.

### Growth performances

Table 5 lists the effects of four different mushroom waster medium or stalk residues on growth performance in broilers at 35 days. The feed intake and body weight gain of the CMPE and CMPS groups were lower than other groups (p<0.05) from days 1 to 21. In the late feeding period (22 to 35 d), the CMPE group had the best FCR and the lowest feed intake. The group CMWM had the highest body weight gain (p<0.05), and the CMPE group had the lowest feed intake and the best FCR (p<0.05) from days 1 to 35.

### Microbial population in ileum and ceca

Table 6 lists the effects of four different mushroom waster medium or stalk residues on the microbial population in ileum and ceca of broilers. The group CMFV was increased by 0.2 and 0.16 log CFU/g in the ileum and cecum, respectively, for lactic acid bacteria, but there was no significant difference in general. The group CMWM reduced the specific microbial in ileum and cecum at 0.48 and 0.18 log CFU/g, respectively, compared with the control group of E. coli, but there was still no significant difference.

### Intestinal morphology

Table 7 lists the effects of four different mushroom waster medium or stalk residues on the intestinal morphology of broilers. The CMPE and the CMFV groups reduced the depth of jejunum crypt compared with the control group, while the group CMWM had significantly reduced jejunum crypt depth than the other groups. In the results of jejunum villus height and crypt depth ratio, the CMWM and the CMPE groups were significantly higher than the control group.

### The mRNA expression level

**Figure 2:**
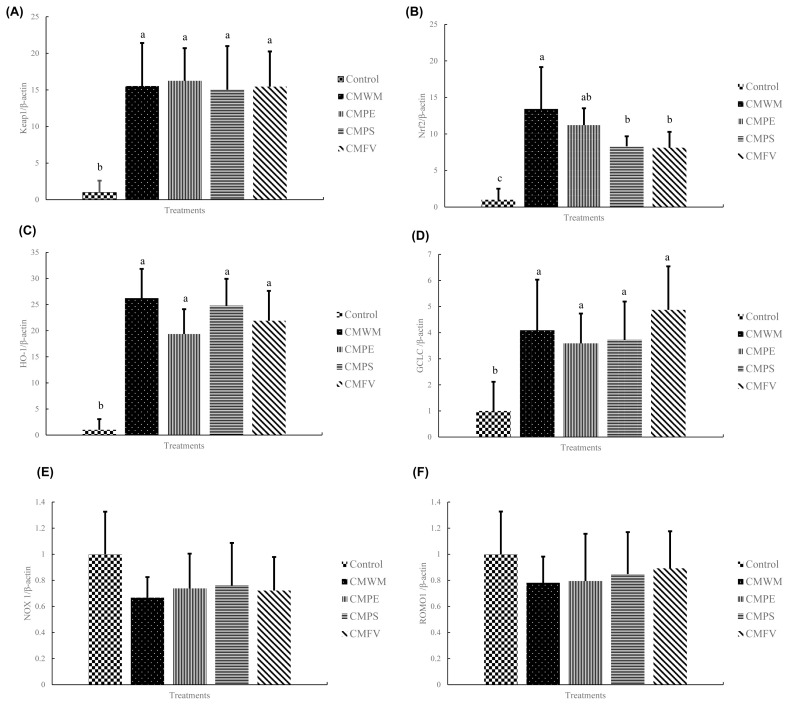
The expression levels of antioxidant-regulated mRNA in peripheral blood mononuclear cells (A) Keap1; (B) Nrf2; (C) HO-1; (D) GCLC; (E) NOX1; (F) ROMO1. *Keap1*, Kelch like ECH associated protein 1; *Nrf2*, nuclear factor (erythroid-derived 2)-like 2; *HO-1*, heme oxygenase -1; *GCLC*, glutamate cysteine ligase catalytic subunit; *NOX1*, NADPH oxygenase 1; *ROMO1*, reactive oxygen species modulator protein 1; CMWM, 1% *Cordyceps militaris* waster medium; CMPE, 0.5% *Cordyceps militaris* waster medium+0.5% *Pleurotus eryngii* stalk residues; CMPS, 0.5% *Cordyceps militaris* waster medium+0.5% *Pleurotus sajor-caju* stalk residues; CMFV, 0.5% *Cordyceps militaris* waster medium+0.5% *Fammulina velutipes* stalk residues. ^a–c^ Means within the same row without common superscripts differ significantly.

Figure 2 illustrates the results of the mRNA expression level in chicken PBMCs. Compared with the control group, the mRNA expressions of Kelch like ECH associated protein 1 (Keap1), nuclear factor (erythroid-derived 2)-like 2 (Nrf2), heme oxygenase-1 (HO-1), and glutamate-cysteine ligase catalytic (GCLC) in the treatment groups were significantly higher. In the mRNA expression of Nrf2, the group CMWM was significantly higher than the group CMPS and the CMFV group. There were no significant differences between the groups in NOX-1, ROMO-1, and immunomodulatory genes.

## DISCUSSION

Animals are typically subjected to a variety of stimuli that increase oxidative stress in the body. The stress makes some proteins, lipids, and even cell membranes and nucleic acids cause some diseases in farm [24–26]. Oxidative stress incompletely replicates DNA, inducing mutations or lesions [27]. The polysaccharides extracted from *Cordyceps militaris* have also confirmed to have free radical scavenging activity [28]. The *Cordyceps militaris*, *Pleurotus eryngii*, *Pleurotus sajor-caju*, and *Fammulina velutipes* are common mushrooms in Asia. These mushrooms have many functional ingredients, such as phenolic compounds, ergosterols, triterpenes, adenosine, flavonoids, polysaccharides, etc. These functional ingredients can improve antioxidant capacity [29], and their compositions in the medium and pedicles are similar to the sporocarp.

The results show that mushroom stalk residues improved body weight gain and FCR, especially in the group CMWM. The present study is similar to Han et al [30]. Lipopolysaccharide (LPS) causes the integrity of the gut barrier incomplete and makes the energy support the immune system, causing lower growth performance [31]. Won et al [32] point out that the administration of 1%, 5%, and 10% of ethanolic extracts of cultured mycelia of *Cordyceps militaris* can effectively reduce α-iNOS produced by stimulation of RAW 264.7 cells with LPS. It is speculated that the mushroom stalk residues can improve intestinal health, reduce inflammatory response.

Intestinal epithelial tissue has a various protein structure that blocks exogenous toxins into the body, toxins intruding into the body will cause an immune response such as macrophages deactivation and detoxification [33]. It shows that the integrity of the intestine is very important for the health and growth of animals. In addition to separating *in vitro* and *in vivo*, the gastro intestinal tract (GIT) can also absorb nutrients such as proteins, sugars, lipids, and various vitamins and minerals from the cavity [31]. The CMWM group can increase the villus height and crypt depth ratio of the jejunum, which can increase the surface area of the villus and improve the absorption of nutrients [34]. The deeper the crypt is, the more the intestinal cells proliferate and the more energy is consumed, which is unpleasant in broilers. The intestinal cells will compensate growth to suppress stress, and crypt depth is one of the indicators of whether the intestine is in an abnormal physiological state. The results show that the crypt depth of the CMPS group was lower than the control group. Thus, it can be speculated that mushroom stalk residues have the ability to reduce stress from environment. If fiber in the diet is insufficient, then gut microbiota resorts to host-secreted mucus glycoproteins as a nutrient source, leading to erosion of the colonic mucus barrier [35]. The mushroom stalk residues can increase the fiber content in feed, reduce erosion of intestinal mucin glycoprotein by microbial, and indirectly reduce stress in the intestine.

There are specific microbes that are stable in the intestines, which have a very complex microbial ecology especially in the cecum [36]. The intestinal microbes can be important in the health of the host. In order to maintain health, it is necessary to improve the intestinal microbes. In recent years, probiotics or prebiotics have been used to improve intestinal microbes [36,37]. It was found that the mushroom stalk residues could increase the number of *Lactobacillus* spp. and reduce *Coliform* amount in the data, but there are no significant differences in each group. It is similar to the experiment result of feeding broiler chickens with *Cordyceps* water extract that promoted the growth of *Lacribacillus* and reduced *Salmonella* and *Escherichia coli* in the intestine [38]. It may be due to the antibacterial activity of some polysaccharides that are not easily absorbed and can improve the intestinal health [36,39,40].

There is a dynamic redox system in animals. Free radicals or ROS are generated during normal metabolism. There are two kinds of antioxidant systems in the cells in order to remove these oxidizing substances: i) a non-enzymatic system and ii) an enzymatic system. The former includes glutathione, which can directly remove ROS, and the latter (including catalase) can reduce hydrogen peroxide to hydrogen oxide [25]. The anti-oxidation pathway of Nrf2/Keap1 belongs to the non-enzymatic system. There is no activity when Nrf2 is bound with Keap1 in the cytoplasm. Keap1 will be separated from Nrf2 after phosphating. Nrf2 will enter the nucleus and form a dimer with Maf protein and will react with the antioxidant responsive element and regulate antioxidant genes such as *GCLC* and *HO-1* [41]. Glutamate cysteine ligase consists of heavy chain GCLC and light chain glutamate-cysteine ligase modulatory; among them, GCLC is the site of catalytic antioxidant activity [42]. HO-1 is the rate-limiting enzyme of heme metabolism, which can catalyze the cleavage of heme into biliverdin (BV) and further be converted into bilirubin (BR) by biliverdin reductase on cytoplasmic [43]. The cycles of BR and BV can reduce the risk of excessive H_2_O_2_ on animals [44]. The NADPH oxygenase 1 (NOX1) family and reactive oxygen species modulator protein 1 (ROMO1) are proteins that are produced in normal physiology. The former is NADPH oxidase, and the latter is a protein produced in the electron transport chain. NOX-1 causes some lipid peroxidation and even endothelial damage [45]. ROMO1 is a regulator of ROS in mitochondria, which causes oxidative stress and apoptosis of cells [46]. The results show that the mushroom stalk residues can enhance the Nrf2 pathway, probably because phenols can activate Nrf2 [47], but NOX1 and ROMO1 exhibit no significant difference.

## CONCLUSION

Based on the above results, CMWM (1%) has the best effect on improving intestinal health and growth performance of broilers and can enhance the antioxidant capacity through the Nrf2 and Keap1 pathways in order to show resistance to stress compare with other groups. The *Cordyceps militaris* waster medium has the potential to be developed as a feed additive in the future.

## Figures and Tables

**Table 1:** Composition and calculated analysis (g/kg as fed) of the basal diet for broilers (1 to 21 days)

Items	Control1)	CMWM1)	CMPE1)	CMPS1)	CMFV1)
Ingredients	----------------------------------------------------------------------- g/kg ----------------------------------------------------------------------				
Yellow corn	488.7	472.3	474.7	474.9	473.3
Soybean meal (CP 44.0%)	345.2	330.0	314.5	330.1	332.0
Full fat soybean meal	83.6	103.2	120.0	100.7	100.0
Soybean oil	35.3	37.3	33.6	37.1	37.5
Calcium carbonate	16.2	16.2	16.2	16.2	16.2
Monocalcium phosphate	18.6	18.6	18.6	18.6	18.6
DL-methionine	2.0	2.0	2.0	2.0	2.0
L-lysine-HCl	3.7	3.7	3.7	3.7	3.7
NaCl	3.9	3.9	3.9	3.9	3.9
Choline-Cl (50%)	0.8	0.8	0.8	0.8	0.8
Vitamin premix 2)	1.0	1.0	1.0	1.0	1.0
Mineral premix 3)	1.0	1.0	1.0	1.0	1.0
CM	-	10.0	5.0	5.0	5.0
PE	-	-	5.0	-	-
PS	-	-	-	5.0	-
FV	-	-	-	-	5.0
Total	1,000	1,000	1,000	1,000	1,000
Calculated nutrient value					
ME (kcal/kg)	3,050.0	3,050.0	3,050.0	3,050.1	3,050.1
CP (% DM)	23.0	23.0	23.0	23.0	23.0
Calcium (% DM)	1.05	1.05	1.05	1.05	1.05
Total phosphorus (% DM)	0.76	0.76	0.76	0.76	0.76
Available phosphorus, (% DM)	0.52	0.52	0.52	0.52	0.52
Lysine (% DM)	1.54	1.54	1.54	1.54	1.54
Methionine+cystein (% DM)	0.90	0.90	0.90	0.90	0.90
Analyzed nutrient value					
Crude protein (% DM)	23.0	22.9	22.9	23.1	23.2
Crude fat (% DM)	6.6	6.8	7.1	7.2	6.9

CP, crude protein; CM, *Cordyceps militaris* waster medium; PE, *Pleurotus eryngii* stalk residues; PS, *Pleurotus sajor-caju* stalk residues; FV, *Fammulina velutipes* stalk residues; CP, crude protein; ME, metabolizable energy; DM, dry matter.

1) Control, basal diet; CMWM, basal diet supplemented with 1% CM; CMPE, basal diet supplemented with 0.5% CM+0.5% PE; CMPS, basal diet supplemented with 0.5% CM+0.5% PS; CMFV, basal diet supplemented with 0.5% CM+0.5% FV.

2) Supplied per kg of diet: Vit A 15,000 U; Vit D_3_ 3,000 U; Vit E 30 mg; Vit K_3_ 4 mg; riboflavin 8 mg; pyridoxine 5 mg; Vit B_12_ 25 μg; Ca-pantothenate 19 mg; niacin 50 mg; folic acid 1.5 mg; biotin 60 μg.

3) Supplied per kg of diet: Co 0.255 mg; Cu 10.8 mg; Fe 90 mg; Zn 68.4 mg; Mn 90 mg; Se0.18 mg.

**Table 2:** Composition and calculated analysis (g/kg as fed) of the basal diet for broilers (22 to 35 days)

Items	Control1)	CMWM1)	CMPE1)	CMPS1)	CMFV1)
Ingredient	--------------------------------------------------- g/kg ---------------------------------------------------				
Yellow corn	536.0	522.8	523.0	523.0	523.0
Soybean meal (CP 44.0%)	280.8	263.8	291.4	291.5	291.5
Full fat soybean meal	100.0	117.3	86.0	86.0	86.0
Soybean oil	42.1	45.0	48.5	48.4	48.4
Calcium carbonate	13.4	13.4	13.4	13.4	13.4
Monocalcium phosphate	16.6	16.6	16.6	16.6	16.6
DL-methionine	1.3	1.3	1.3	1.3	1.3
L-lysine-HCl	3.2	3.2	3.2	3.2	3.2
NaCl	3.8	3.8	3.8	3.8	3.8
Choline-Cl (50%)	0.8	0.8	0.8	0.8	0.8
Vitamin premix2)	1.0	1.0	1.0	1.0	1.0
Mineral premix3)	1.0	1.0	1.0	1.0	1.0
CM	-	10.0	5.0	5.0	5.0
PE	-	-	5.0	-	-
PS	-	-	-	5.0	-
FV	-	-	-	-	5.0
Total	1,000	1,000	1,000	1,000	1,000
Calculated nutrient value					
ME (kcal/kg)	3,173.63	3,173.65	3,173.63	3,173.63	3,173.64
Crude protein (% DM)	21.01	21.01	21.01	21.01	20.01
Calcium (% DM)	0.89	0.89	0.89	0.89	0.89
Total phosphorus (% DM)	0.70	0.70	0.70	0.70	0.70
Available phosphorus (% DM)	0.47	0.47	0.47	0.47	0.47
Lysine (% DM)	1.38	1.38	1.37	1.37	1.37
Methionine+cysteine (% DM)	0.79	0.78	0.78	0.78	0.78
Analyzed nutrient value					
Crude protein (% DM)	21.1	20.9	21	21.2	21.1
Crude fat (% DM)	8.9	9.2	9.3	9.4	8.7

CM, *Cordyceps militaris* waster medium; PE, *Pleurotus eryngii* stalk residues; PS, *Pleurotus sajor-caju* stalk residues; FV, *Fammulina velutipes* stalk residues; CP, crude protein; ME, metabolizable energy; DM, dry matter.

1) Control, basal diet; CMWM, basal diet supplemented with 1% CM; CMPE, basal diet supplemented with 0.5% CM+0.5% PE; CMPS, basal diet supplemented with 0.5% CM+0.5% PS; CMFV, basal diet supplemented with 0.5% CM+0.5% FV.

2) Supplied per kg of diet: Vit A 15,000 U; Vit D_3_ 3,000 U; Vit E 30 mg; Vit K_3_ 4 mg; riboflavin 8 mg; pyridoxine 5 mg; Vit B_12_ 25 μg; Ca-pantothenate 19 mg; niacin 50 mg; folic acid 1.5 mg; biotin 60 μg.

3) Supplied per kg of diet: Co 0.255 mg; Cu 10.8 mg; Fe 90 mg; Zn 68.4 mg; Mn 90 mg; Se 0.18 mg.

**Table 3:** Primers used for q-PCR analysis

Gene	Forward primer (from 5′ to 3′) Reverse primer (from 5′ to 3′)	PCR product size (bp)	Accession in GenBank
*β-actin*	CTGGCACCTAGCACAATGAA	109	X00182.1
	ACATCTGCTGGAAGGTGGAC		
*Keap1*	ATGGCCACACTTTTCTGGAC	137	AB020063.1
	ATCAATTTGCTTCCGACAGG		
*Nrf2*	GGAAGAAGGTGCGTTTCGGAGC	171	NM_205117.1
	GGGCAAGGCAGATCTCTTCCAA		
*HO-1*	AGCTTCGCACAAGGAGTGTT	106	X56201.1
	GGAGAGGTGGTCAGCATGTC		
*GCLC*	CAGCACCCAGACTACAAGCA	118	XM_419910.3
	CTACCCCCAACAGTTCTGGA		
*NOX1*	CAATGCAGCACTCCACTTTG	185	NM_001101830.1
	GACAAGATCTCCGCAAGACC		
*ROMO1*	AGCCCAGCTGCTTCGACAGAGT	115	NM_001198821.1
	CGTCCTCTCATGCCGATCCTGA		

q-PCR, quantitative polymerase chain reaction; *Keap1*, Kelch like ECH associated protein 1; *Nrf2*, nuclear factor (erythroid-derived 2)-like 2; *HO-1*, heme oxygenase -1; *GCLC*, glutamate cysteine ligase catalytic subunit; *NOX1*, NADPH oxygenase 1; *ROMO1*, reactive oxygen species modulator protein 1.

**Table 4:** Chemical composition and content of the bioactive compounds of mushroom stalk residues

Items	Stalk residues			

	CM	PE	PS	FV
Chemical composition				
Dry matter (%)	97.3±0.3	94.5±0.3	97.9±0.3	93.0±0.4
Ash (% DM)	1.2±0.02	5.9±0.01	6.8±0.01	11.5±0.01
Crude protein (%DM)	7.8±0.08	17.2±0.11	22.4±0.1	12.7±0.08
NDF (%DM)	63.1±1.8	57.9±1.5	61.9±1.4	60.8±1.0
ADF (%DM)	8.0±0.35	7.4±0.25	8.2±0.41	8.0±0.65
Crude fat (%DM)	3.8±0.49	6.4±0.54	2.0±0.39	5.0±0.57
Bioactive compounds				
Polysaccharide (mg/g)	72.4±3.6	8.2±2.2	8.8±2.3	5.7±0.3
Total triterpenes (mg/g)	1.2±0.1	3.1±0.7	3.9±1.1	6.1±0.5
Total phenolics (mg GAE/g DW)	7.1±0.6	14.9±1.0	9.8±0.3	18.1±0.5
Total Flavonoid (mg QE/g DW)	7.1±0.3	10.6±0.3	4.7±0.3	13.8±0.1

The value is expressed as the mean±standard deviation (n = 5).

CM, *Cordyceps militaris* waster medium; PE, *Pleurotus eryngii* stalk residues; PS, *Pleurotus sajor-caju* stalk residues; FV, *Fammulina velutipes* stalk residues; DM, dry matter; GAE, gallic acid equivalent; DW, dry weight; QE, quercetin equivalent.

**Table 5:** Effects of mushroom stalk residues supplemented in diet on growth performance of broilers

Items	Treatment1)					SEM	p-value

	Control	CMWM	CMPE	CMPS	CMFV		
1–21 d							
Weight gain (kg)	0.799^a^	0.818^a^	0.742^b^	0.734^b^	0.790^a^	0.005	<0.001
Feed consumption (kg)	0.981^ab^	0.938^ab^	0.920^b^	0.926^b^	1.018^a^	0.041	0.103
FCR	1.227	1.149	1.239	1.261	1.291	0.018	0.190
22–35 d							
Weight gain (kg)	1.115	1.125	1.124	1.117	1.112	0.009	0.975
Feed consumption (kg)	1.758^a^	1.759^a^	1.588^b^	1.648^ab^	1.769^a^	0.017	0.042
FCR	1.578^a^	1.567^a^	1.414^b^	1.531^ab^	1.572^a^	0.020	0.095
1–35 d							
Weight gain (kg)	1.872^ab^	1.900^a^	1.824^b^	1.822^b^	1.861^ab^	0.009	0.022
Feed consumption (kg)	2.739^ab^	2.697^ab^	2.508^c^	2.574^bc^	2.787^a^	0.024	0.040
FCR	1.463^ab^	1.419^ab^	1.376^b^	1.413^ab^	1.498^a^	0.014	0.161

SEM, standard error of the mean; FCR, feed conversion rate.

1) Control, basic feed; CMWM, 1% *Cordyceps militaris* waster medium; CMPE, 0.5% *Cordyceps militaris* waster medium+0.5% *Pleurotus eryngii* stalk residues; CMPS, 0.5% *Cordyceps militaris* waster medium+0.5% *Pleurotus sajor-caju* stalk residues; CMFV, 0.5% *Cordyceps militaris* waster medium+0.5% *Fammulina velutipes* stalk residues.

a–c Means within the same row without common superscripts differ significantly (p<0.05).

**Table 6:** Effects of mushroom stalk residues supplemented in diet on microbial parameter in intestinal content of broilers (day 35)

Microbial parameter (log CFU/g)	Treatment1)					SEM	p-value

	Control	CMWM	CMPE	CMPS	CMFV		
*Lactobacillus* spp.							
Ileum	7.03	7.19	7.02	7.15	7.23	0.042	0.442
Caecum	8.33	8.48	8.37	8.35	8.49	0.026	0.198
Coliform							
Ileum	7.47	6.99	7.20	7.00	7.18	0.029	0.339
Caecum	9.64	9.46	9.60	9.49	9.47	0.024	0.126
*Clostridium perfringens*							
Ileum	6.93	6.75	7.15	7.07	7.05	0.060	0.332
Caecum	9.19	9.05	9.30	8.88	8.87	0.027	0.361

SEM, standard error of the mean; CFU, colony-forming unit.

1) Control, basic feed; CMWM, 1% *Cordyceps militaris* waster medium; CMPE, 0.5% *Cordyceps militaris* waster medium+0.5% *Pleurotus eryngii* stalk residues; CMPS, 0.5% *Cordyceps militaris* waster medium+0.5% *Pleurotus sajor-caju* stalk residues; CMFV, 0.5% *Cordyceps militaris* waster medium+0.5% *Fammulina velutipes* stalk residues.

**Table 7:** Effects of mushroom stalk residues supplemented in diet on intestinal morphology of broilers (day 35)

Items	Treatment1)					SEM	p-value

	Control	CMWM	CMPE	CMPS	CMFV		
Jejunum							
Villus height (μm)	1,237.1	1,240.3	1,323.4	1,343.9	1,292.1	19.4	0.300
Crypt depth (μm)	214.5^a^	177.1^c^	188.9^bc^	203.7^ab^	193.8^bc^	2.67	<0.01
Tunica muscularis (μm)	262.8	247.4	246.0	259.8	264.1	3.74	0.372
Villus:crypt	6.18^b^	7.40^a^	7.21^a^	6.81^ab^	6.99^ab^	0.12	0.03
Ileum							
Villus height (μm)	1,040.9	994.5	993.6	1,018.7	997.1	9.55	0.447
Crypt depth (μm)	167.2	153.2	170.8	155.9	168.3	2.97	0.228
Tunica muscularis (μm)	279.7	262.3	267.5	260.7	264.6	4.78	0.737
Villus:crypt	6.52	6.94	6.19	6.77	6.08	0.13	0.167

SEM, standard error of the mean.

1) Control, basic feed; CMWM, 1% *Cordyceps militaris* waster medium; CMPE, 0.5% *Cordyceps militaris* waster medium+0.5% *Pleurotus eryngii* stalk residues; CMPS, 0.5% *Cordyceps militaris* waster medium+0.5% *Pleurotus sajor-caju* stalk residues; CMFV, 0.5% *Cordyceps militaris* waster medium+0.5% *Fammulina velutipes* stalk residues.

a–c Means within the same row without common superscripts differ significantly (p<0.05).
